# The chemotherapeutic drug carboplatin affects macrophage responses to LPS and LPS tolerance via epigenetic modifications

**DOI:** 10.1038/s41598-021-00955-7

**Published:** 2021-11-03

**Authors:** Atsadang Boonmee, Salisa Benjaskulluecha, Patipark Kueanjinda, Benjawan Wongprom, Thitiporn Pattarakankul, Tanapat Palaga

**Affiliations:** 1 Department of Microbiology, Faculty of Science, Chulalongkorn University, Bangkok, Thailand; 2 Center of Excellence in Immunology and Immune-Mediated Diseases, Chulalongkorn University, Bangkok, Thailand; 3 Inter-Disciplinary Graduate Program in Medical Microbiology, Graduate School, Chulalongkorn University, Bangkok, Thailand; 4 Department of Microbiology, Faculty of Medicine, Chulalongkorn University, Bangkok, Thailand

**Keywords:** Drug discovery, Biochemistry, Cytokines, Cancer, Cancer therapy, Immunology, Cytokines, Innate immunity

## Abstract

Following re-exposure to lipopolysaccharide (LPS), macrophages exhibit an immunosuppressive state known as LPS tolerance, which is characterized by repressed proinflammatory cytokine production. LPS-induced tolerance in macrophages is mediated in part by epigenetic changes. Carboplatin, an anticancer chemotherapeutic drug, exerts its effect by inhibiting DNA replication and transcription, as well as through epigenetic modifications. Through an unbiased screen, we found that carboplatin rescued TNF-α and IL-6 production in LPS-tolerant macrophages. Transcriptomic analysis and gene set enrichment analyses revealed that p53 was one of the most significantly upregulated hallmarks in both LPS-primed and LPS-tolerant macrophages in the presence of carboplatin, while E2F and G2/M were the most negatively regulated hallmarks. Heterochromatin protein 1 (HP1-α), which is associated with gene silencing, was significantly reduced in carboplatin-treated LPS-tolerant macrophages at the mRNA and protein levels. Dynamic changes in the mRNA level of genes encoding H3K9me3 methyltransferases, *setdb2, kdm4d*, and *suv39h1* were induced in the presence of carboplatin in LPS-tolerant macrophages. Taken together, we provide evidence that carboplatin treatment interferes with proinflammatory cytokine production during the acute LPS response and LPS tolerance in macrophages, possibly via H3K9me3 modification.

## Introduction

Carboplatin, an antineoplastic drug, is a platinum-based agent classified as an alkylating agent^1^. Its mode of action is mediated by various mechanisms including (1) transferring alkyl groups to the guanine residues of DNA, resulting in DNA fragmentation and the formation of mispaired bases; and (2) forming either inter or intra-strand crosslinks, which causes DNA damage and prevents strand separation during DNA synthesis or transcription^2,3^. Platinum-based compounds, including cisplatin and carboplatin, are the most commonly used treatments for solid tumors, such as ovarian cancer^4^. Carboplatin is known to be more effective and has several toxicological advantages over cisplatin^5^; it exerts its antitumor effects by forming DNA adducts and subsequently inhibiting DNA replication and transcription.

Epigenetic modifications are crucial for normal development and homeostatic maintenance of tissue-specific gene expression in mammalian cells^6^. Interference of epigenetic modification patterns can alter gene expression, causing cells to undergo malignant cellular transformation, leading to abnormal function. It has been shown that global changes in the epigenetic landscape is a hallmark of cancer. A number of studies have reported on anticancer drugs that are linked to epigenetic machinery^7^. Carboplatin has been used to treat various cancers, including testicular, ovarian, brain, bladder, and lung cancers^8–12^. Furthermore, a previous study also showed that carboplatin treatment induces trimethylation of histone H3 lysine 4 (H3K4) and acetylation of histone H3 lysine 9 (H3K9) at reactivated genes of YB5 cells, a cell line derived from SW48 colon cancer cells^13^. These observations point to the potential interference of carboplatin with cellular epigenetic machinery.

It has long been believed that immune memory exists only as a component of adaptive immunity; however, this concept has been revised recently^14^. Traditionally, innate immune responses are thought to be nonspecific and without the capability to adapt. In contrast to the innate immune response, the adaptive immune response to pathogens is specific and able to produce long-lived immunological memory^15^. Emerging evidence suggests that innate immune cells (e.g., macrophages, monocytes, and natural killer cells) are also able to develop memory-like responses to previous encounters against bacterial lipopolysaccharides (LPS)^16^. This innate immune memory manifests as a repressed response known as tolerance, which is postulated to be a compensatory mechanism that helps limit the possible harm caused by an overwhelming immune cell response to stimuli in a successive second exposure. On the other hand, the memory-dependent enhanced response, called trained immunity, aims to improve tissue surveillance and protect against tissue damage caused by the hyperreaction of repeated or chronic infections^17,18^.

It has been reported that tolerance mechanisms involve systemic inflammation resulting in immune paralysis, which has often been found in sepsis patients and leads to secondary infection that can be lethal^19^. To date, increasing evidence of the tolerance mechanisms in sepsis has been linked to epigenetic modifications, including DNA methylation and histone modification^20^. Furthermore, epigenetic changes in LPS-induced tolerant macrophages have been elucidated through histone acetylation and methylation profiling^21^. Certain studies have reported that the processes of inflammation and infection are regulated in part by histone methylation^22^. Methylation of histone H3 at different amino acid side chains has a profound impact on the transcription of genes in the vicinity. H3K4me3 is a representative active histone mark in the promoter of active genes, while H3K9me3 is associated with gene silencing. Thus, inhibition of this process by dampening methyltransferase enzymes (HMTs) or increasing histone demethylase enzymes (KDMs) is a promising adjunct therapy for immune tolerance-mediated diseases.

In this study, we uncovered the effect of carboplatin on the response to LPS and LPS tolerance in macrophages. Based on our detailed study, we proposed that carboplatin might be able to interfere with epigenetic controls, especially alterations in the H3K9 hallmark, and thereby alters responses in LPS-induced tolerant macrophages. This knowledge will allow for a better understanding of the effect of antitumor drugs on innate immune memory, which may have implications for the clinical care of chemotherapy-treated cancer patients.

## Results

### Carboplatin restored LPS-induced production of TNF-α and IL-6 in tolerant macrophages

**Figure 1:**
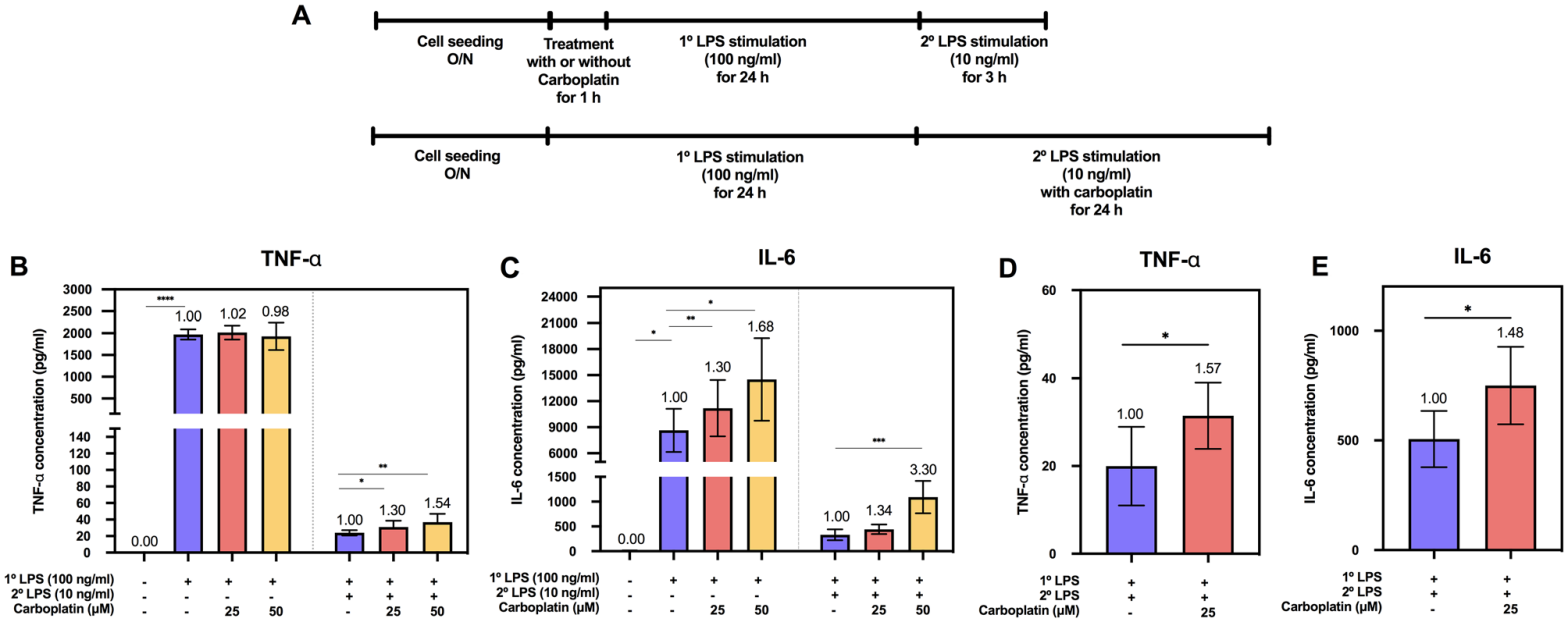
Effect of carboplatin on TNF-α and IL-6 production in LPS-primed and LPS-tolerant macrophages. (**A**) Schematic of the procedures for carboplatin and LPS treatment. BMDMs were pretreated with or without carboplatin (25 or 50 μM) for 1 h prior to LPS priming (1°LPS; 100 ng/ml) for 24 h. After 24 h incubation, cells were challenged with LPS (2°LPS; 10 ng/ml) for 3 h (upper panel) or 24 h (lower panel). (**B**–**E**) TNF-α (**B**,**D**) and IL-6 (**C**,**E**) production after 3 h or 24 h of incubation was measured by ELISA. Data are representative of at least 3 independent experiments. *, **, ***, **** indicate *p* < 0.05, *p* < 0.01, *p* < 0.001, *p* < 0.0001, respectively, using one-way ANOVA. The numbers above the bars indicate fold differences compared to those without carboplatin.

First, we tested the procedure to induce LPS tolerance in bone marrow-derived macrophages (BMDMs); we monitored the levels of the proinflammatory cytokines TNF-α and IL-6 in BMDMs that were primed with LPS (1° LPS; 100 ng/ml) without carboplatin for 24 h and compared them with BMDMs receiving 1° LPS followed by re-exposure to LPS (2° LPS; 10 ng/ml) for another 3 h (Fig. 1A, upper panel). As expected for the repressed response, the levels of both cytokines dramatically decreased in the tolerant cells compared to the cells primed with a single LPS exposure (Fig. 1B,C and Supplementary Fig. S1). The fold changes were reduced by 21.7- and 1.5-fold for TNF-α and IL-6, respectively. As a result, priming BMDMs with LPS at 100 ng/ml, followed by re-exposure to LPS at 10 ng/ml, clearly induced a tolerized response for TNF-α and IL-6 in BMDMs.

We next determined whether carboplatin affected the primary response to LPS in BMDMs by pretreating cells with or without carboplatin at 25 or 50 µM for 1 h prior to LPS stimulation for 24 h (Fig. 1A, upper panel). Compared to the unstimulated control, the LPS-primed cells had significantly increased cytokine production, as shown in Fig. 1B,C (*p* < 0.05). Notably, a statistically significant difference was not observed in the carboplatin-treated cells in the primed state for TNF-α production, but carboplatin treatment significantly increased the level of IL-6 (Fig. 1C).

We further investigated the effects of carboplatin on LPS-induced tolerance in macrophages. Pretreatment of BMDMs with carboplatin during priming with LPS enhanced TNF-α and IL-6 production compared to that of the untreated control in a dose-dependent manner (Fig. 1B,C and Supplementary Fig. S2) (*p* < 0.05). Next, we asked whether carboplatin also has an effect when added during re-exposure to LPS. A scheme of the procedure is shown in Fig. 1A, lower panel. Carboplatin-treated cells showed significantly higher levels of TNF-α and IL-6 production than vehicle control-treated cells (*p* < 0.05) (Fig. 1D,E). These results indicated that carboplatin reduces LPS-induced tolerance when added during the LPS priming step or the LPS restimulation step.

**Figure 2:**
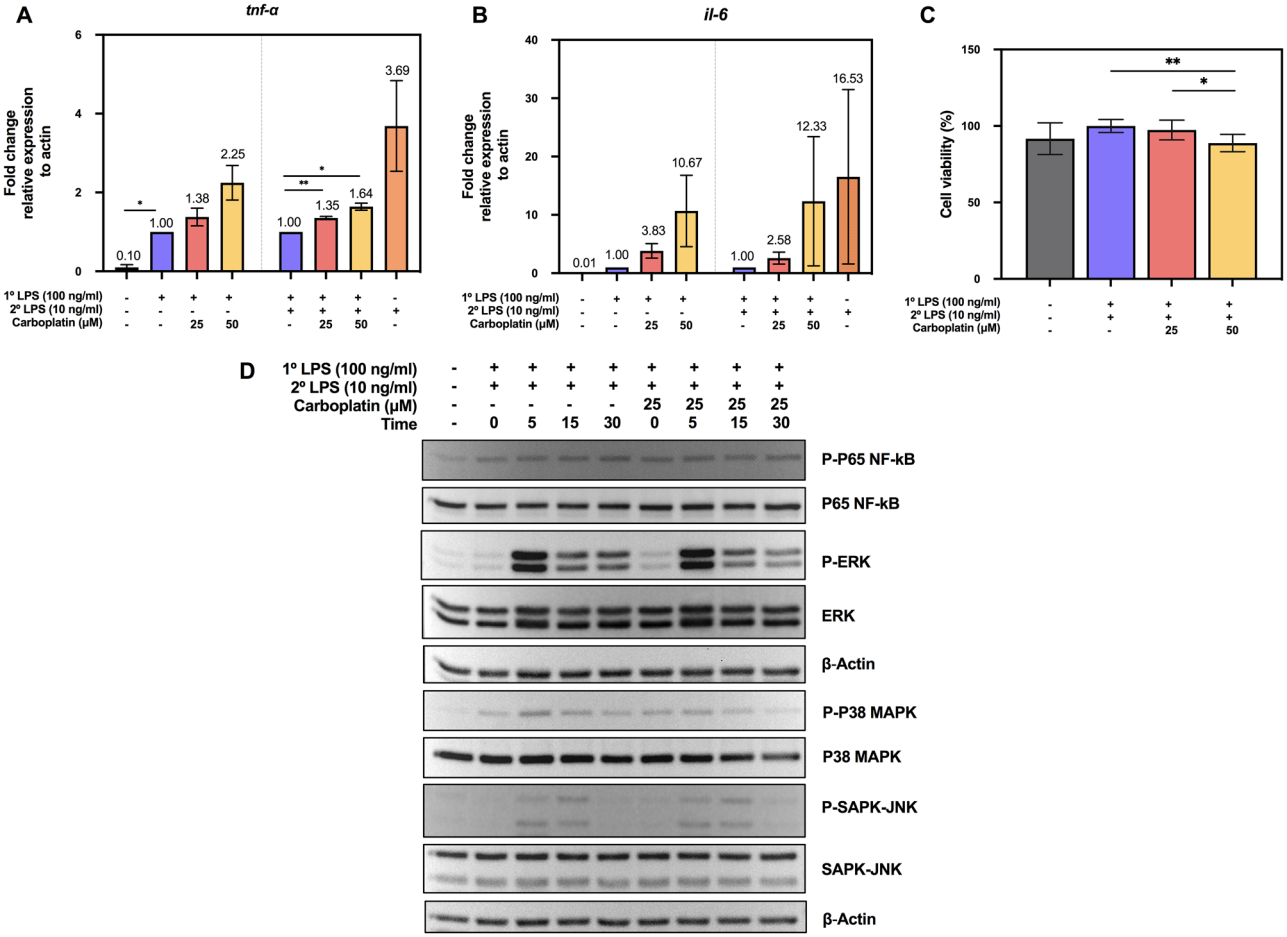
Effects of carboplatin on the transcription of *tnf-*α and *il-6* and the TLR4 signaling pathway. (**A**,**B**) Total RNA from LPS-primed and LPS-tolerant BMDMs with or without carboplatin as described in Fig. 1 was subjected to RT-qPCR for *tnf-α* (**A**) and *il-6* (**B**). Data are representative of 3 biological replicates. *, ** indicate *p* < 0.05 and 0.01, respectively, using one-way ANOVA. The numbers above the bars indicate fold differences compared to the cells without carboplatin. (**C**) Cell viability of LPS-tolerant macrophages in the presence or absence of carboplatin for 24 h was measured using an MTT assay. (**D**) Downstream TLR-4 signaling pathway activity in LPS-primed and LPS-tolerant macrophages with or without carboplatin was detected by western blotting. Actin and total targeted proteins were used as loading controls for each phosphorylated protein. The data shown are representative blots of replicates (n = 3).

We then confirmed this phenomenon at the transcript level using qPCR followed the procedure scheme in Fig. 1A, upper panel. As shown in Fig. 2A,B, incubation of macrophages in the presence of carboplatin (25 or 50 µM) for 24 h increased the mRNA levels of both *tnf-α* and *il-6* during the LPS priming step and LPS restimulation in a dose-dependent manner (*p* < 0.05).

Next, we tested whether carboplatin treatment induced cytotoxicity in BMDMs using a tetrazolium dye 3-(4,5-dimethylthiazol-2-yl)-2,5-diphenyltetrazolium (MTT) assay. BMDMs were treated with 25 or 50 µM carboplatin and stimulated with LPS (100 ng/ml) for 24 h, followed by restimulation with LPS for another 24 h. We observed that 25 µM carboplatin treatment did not induce cytotoxicity, and the cell viability remained unaltered compared to the control (Fig. 2C). However, cell viability was slightly decreased when using 50 µM carboplatin. Thus, 25 µM carboplatin was used in further experiments.

### Carboplatin did not interfere with the TLR4 signaling pathway in LPS-tolerant macrophages

Stimulation of Toll-like receptor 4 (TLR4) by LPS activates the release of key proinflammatory cytokines that are essential for potent immune responses. Exposure to LPS leads to TLR4-dependent stimulation of the NF-κB, MAPK, ERK and SAPK-JNK pathways. Therefore, we first asked whether the effect of carboplatin on proinflammatory cytokines in LPS-tolerant macrophages involved signaling immediately downstream of TLR4. BMDM cell lysates were harvested after priming with LPS in the presence or absence of carboplatin and re-exposing to secondary LPS stimulation for 0, 5, 15, or 30 min. As shown in Fig. 2D and Supplementary Figs. S3, S4, phosphorylation of NF-κB p65, p-MAPKs, p-ERK and p-SAPK-JNK was readily detected at 5 and 10 min after LPS re-exposure. More importantly, we could not observe any drastic changes in the phosphorylated forms of NF-κB p65, p-MAPKs, p-ERK or p-SAPK-JNK proteins between the control and carboplatin-treated cells (Supplementary Figs. S3, S4). These results suggested that carboplatin treatment did not interfere with the NF-κB or MAPK signaling pathways during LPS-induced tolerance in macrophages.

### Transcriptomic profiles of LPS-tolerant macrophages

We next focused on the transcriptomes of LPS-tolerant macrophages. RNA-Seq transcripts of unstimulated and LPS (100 ng/ml)-primed BMDMs were compared and 975 differentially expressed genes (DEGs) were observed (|log2FC| > 2, *p* < 0.05). Among these, 421 genes were upregulated and 554 genes were downregulated with LPS priming (Supplementary Table S1). The most significantly enriched GO terms (*p* < 0.05) among the upregulated genes included myeloid leukocyte migration, cell chemotaxis, regulation of cell–cell adhesion, leukocyte migration, regulation of inflammatory response, and leukocyte cell–cell adhesion (Supplementary Fig. S5). In contrast, the GO terms associated with downregulated genes included chromosome segregation, nuclear division, nuclear chromosome segregation, mitotic nuclear division, sister chromatid segregation, and organelle fission (Supplementary Fig. S5).

Next, we analyzed DEGs between LPS-stimulated (10 ng/ml) and LPS-tolerant BMDMs. A total of 1,088 genes were differentially expressed in this comparison (|log2FC| > 2, *p* < 0.05). We identified 403 upregulated and 685 downregulated genes in LPS-tolerant macrophages (Supplementary Table S2). Genes that are induced upon LPS re-exposure are defined as nontolerizeable genes, whereas genes that are repressed are termed tolerizeable genes. To understand the physiological roles of the nontolerizeable genes, we performed functional analysis of these genes by GO term enrichment. Leukocyte chemotaxis, myeloid leukocyte migration, response to oxidative stress, cell chemotaxis, leukocyte migration, and granulocyte chemotaxis were identified as enriched GO terms among these genes (Supplementary Fig. S6). This finding indicated that LPS-tolerant macrophages may promote the migration of macrophages.

### DEGs in the presence of carboplatin in LPS-primed macrophages

**Figure 3:**
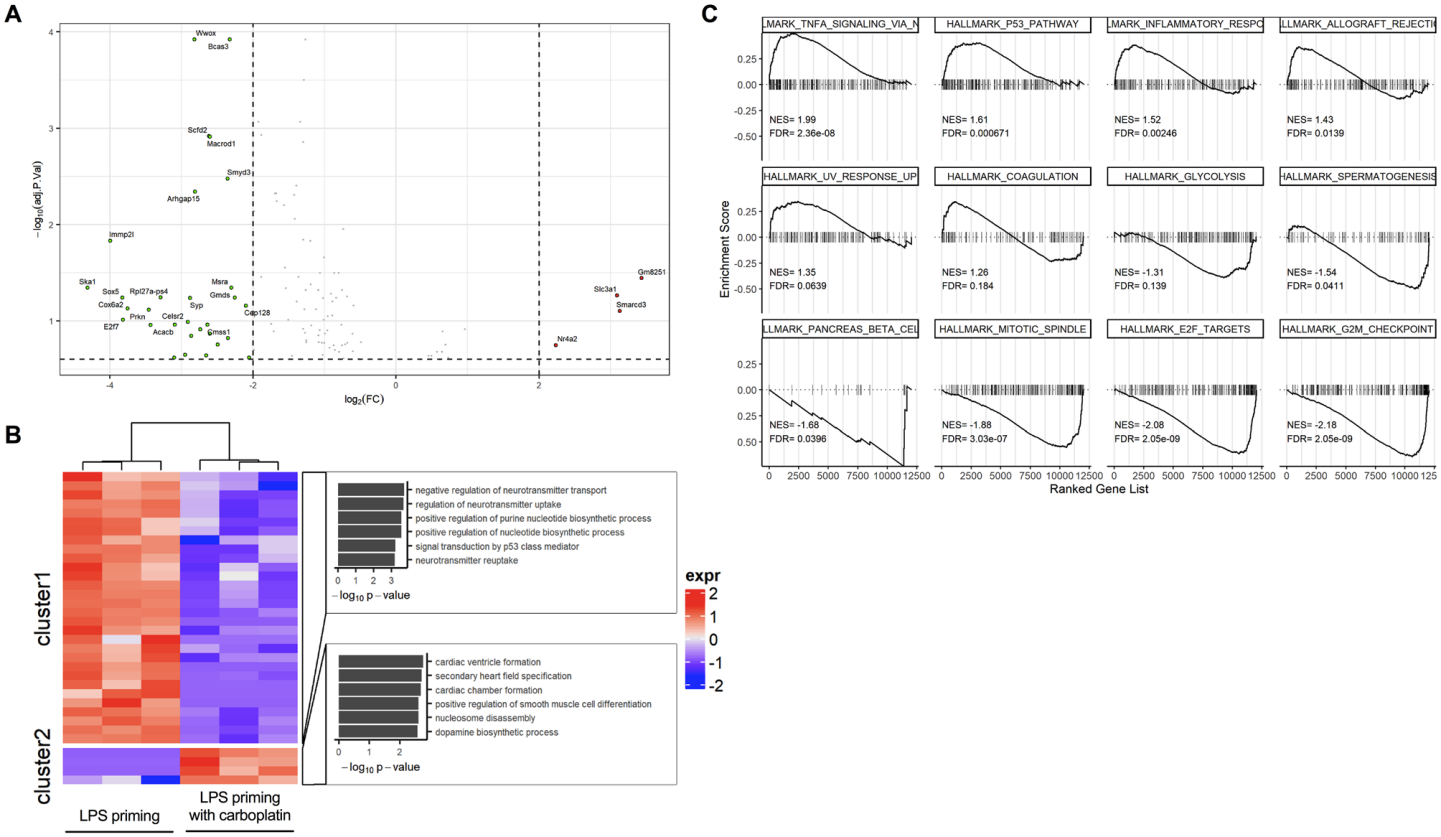
Transcriptomics of LPS-primed macrophages in the presence of carboplatin. (**A**) BMDMs were pretreated with or without carboplatin (25 μM) for 1 h before stimulation with LPS (100 ng/ml) for 24 h. RNA-Seq was performed as described in the “Materials and methods”. Volcano plot showing DEGs of LPS-primed macrophages with or without carboplatin. |log2FC| > 2, *p* < 0.25. (**B**) Heatmap and GO terms associated with DEGs. (**C**) GSEA showing positive and negative hallmark gene sets in LPS-primed macrophages treated with carboplatin.

The effects of carboplatin treatment on LPS-primed macrophages were next investigated. We conducted a comparison between the transcripts of LPS-primed macrophages in the presence or absence of carboplatin. The differentially expressed genes (|log2FC| > 2, *p* < 0.25) are listed in Table 1. Only a few genes were upregulated in the presence of carboplatin in LPS-primed macrophages (Fig. 3A,B). The actin-dependent regulator of chromatin-encoded genes, *smarcd3,* yielded the highest fold change (8-fold), and the *nr4a2* gene encoding the nuclear receptor also showed a significantly increased fold change of 4.72. For downregulated genes, the two transcription factor-encoding genes *sox5* and *e2f7* were differentially downregulated as much as 14-fold in the presence of carboplatin.

**Table 1:** Lists of DEGs in LPS-primed BMDMs in the presence of carboplatin, |log2FC| > 2, *p* < 0.25.

ENSEMBL ID	Gene name	Gene function	logFC	Adj. *p*-val
ENSMUSG00000091844	*Gm8251*	Predicted gene 8251	3.44	0.03589
ENSMUSG00000028949	*Smarcd3*	SWI/SNF related, matrix associated, actin dependent regulator of chromatin, subfamily d	3.13	0.07877
ENSMUSG00000024131	*Slc3a1*	Solute carrier family 3, member 1	3.09	0.05431
ENSMUSG00000026826	*Nr4a2*	Nuclear receptor subfamily 4, group A	2.24	0.17813

ENSEMBL ID	Gene name	Gene function	logFC	Adj. *p*-val
ENSMUSG00000036223	*Ska1*	Spindle and kinetochore associated complex subunit 1	− 4.32	0.04540
ENSMUSG00000056899	*Immp2l*	IMP2 inner mitochondrial membrane peptidase-like (*S. cerevisiae*)	− 4.00	0.01472
ENSMUSG00000041540	*Sox5*	Transcription factor SOX-5	− 3.83	0.05739
ENSMUSG00000020185	*E2f7*	E2F transcription factor 7	− 3.82	0.09730
ENSMUSG00000030785	*Cox6a2*	Cytochrome *c* oxidase subunit 6A2	− 3.75	0.07452
ENSMUSG00000023826	*Prkn*	Parkin RBR E3 ubiquitin protein ligase	− 3.46	0.07675
ENSMUSG00000037196	*Pacrg*	PARK2 coregulated	− 3.43	0.11014
ENSMUSG00000062461	*Rpl27a-ps4*	Ribosomal protein L27A, pseudogene 4	− 3.29	0.05708
ENSMUSG00000039748	*Exo1*	Exonuclease 1	− 3.10	0.24044
ENSMUSG00000042010	*Acacb*	Acetyl-Coenzyme A carboxylase beta	− 3.09	0.10956
ENSMUSG00000031129	*Slc9a9*	Solute carrier family 9 (sodium/hydrogen exchanger), member 9	− 2.95	0.22418
ENSMUSG00000068740	*Celsr2*	Cadherin, EGF LAG seven-pass G-type receptor 2	− 2.91	0.10260
ENSMUSG00000031144	*Syp*	Synaptophysin	− 2.88	0.05760
ENSMUSG00000003992	*Ssbp2*	Single-stranded DNA binding protein 2	− 2.86	0.14330
ENSMUSG00000004637	*Wwox*	WW domain-containing oxidoreductase	− 2.82	0.00012
ENSMUSG00000049744	*Arhgap15*	Rho GTPase activating protein 15	− 2.81	0.00455
ENSMUSG00000029361	*Nos1*	Nitric oxide synthase 1, neuronal	− 2.74	0.12224
ENSMUSG00000052062	*Pard3b*	Par-3 family cell polarity regulator beta	− 2.65	0.22816
ENSMUSG00000022748	*Cmss1*	Cms small ribosomal subunit 1	− 2.64	0.10956
ENSMUSG00000062110	*Scfd2*	Sec1 family domain containing 2	− 2.61	0.00121
ENSMUSG00000064202	*4430402I18Rik*	Spermatogenesis associated 6 like	− 2.60	0.13591
ENSMUSG00000036278	*Macrod1*	Mono-ADP ribosylhydrolase 1	− 2.60	0.00123
ENSMUSG00000063458	*Lrmda*	Leucine rich melanocyte differentiation associated	− 2.49	0.17658
ENSMUSG00000055067	*Smyd3*	SET and MYND domain containing 3	− 2.36	0.00333
ENSMUSG00000074818	*Pdzd7*	PDZ domain containing 7	− 2.35	0.15092
ENSMUSG00000059439	*Bcas3*	BCAS3 microtubule associated cell migration factor	− 2.32	0.00012
ENSMUSG00000054733	*Msra*	Methionine sulfoxide reductase A	− 2.30	0.04540
ENSMUSG00000038372	*Gmds*	GDP-mannose 4,6-dehydratase	− 2.25	0.05739
ENSMUSG00000061533	*Cep128*	Centrosomal protein 128	− 2.10	0.06959
ENSMUSG00000020604	*Arsg*	Arylsulfatase G	− 2.05	0.23980

The effect of carboplatin on associated pathways in LPS priming was analyzed using gene set enrichment analysis (GSEA). TNF signaling, the p53 pathway, and inflammatory response hallmark gene sets were shown to be positively regulated (FDR < 0.05, Fig. 3C). Hallmark gene sets of E2F, the G2/M checkpoint and the mitotic spindle were significantly downregulated (FDR < 0.05, Fig. 3C). Glycolysis hallmark gene sets were also found to be negatively regulated, suggesting that carboplatin has a negative impact on the metabolic switch following LPS stimulation.

### Carboplatin-induced changes in the transcriptomic profiles of LPS-tolerant macrophages

**Figure 4:**
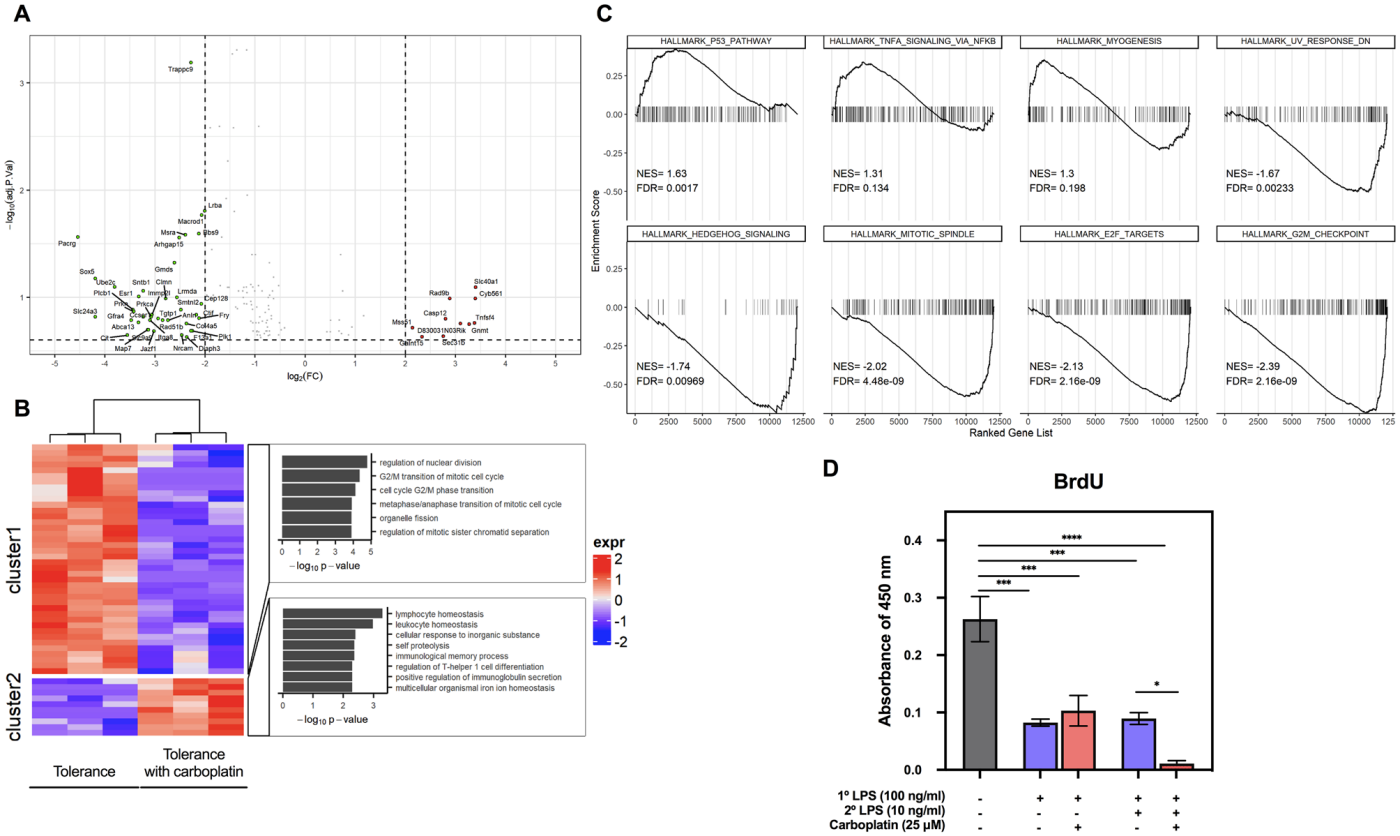
Transcriptomics of LPS-tolerant macrophages in the presence of carboplatin. (**A**) BMDMs were pretreated with or without carboplatin (25 μM) for 1 h before stimulation with LPS (100 ng/ml) for 24 h. The medium was removed, and fresh medium with LPS (10 ng/ml) was added to induce LPS-tolerant macrophages. RNA was harvested at 3 h, and RNA-Seq was performed as described in the “Materials and methods”. Volcano plot showing differentially expressed genes of LPS-induced tolerant macrophages with or without carboplatin. |log2FC| > 2, *p* < 0.25. (**B**) Heatmap and GO terms associated with differentially expressed genes. (**C**) GSEA showing positive and negative hallmark gene sets in LPS-induced tolerant macrophages treated with carboplatin. (**D**) A BrdU incorporation assay was performed using carboplatin-treated LPS-primed and LPS-tolerant macrophages for 24 h. *, **, ***, **** indicate *p* < 0.05, *p* < 0.01, *p* < 0.001, *p* < 0.0001, respectively, using one-way ANOVA.

To gain insights into how carboplatin affects LPS tolerance in macrophages, we performed a comparison between the transcripts of carboplatin-treated and untreated LPS-tolerant macrophages. Fifty DEGs (|log2FC| > 2, *p* < 0.25) were identified (Table 2, Fig. 4A,B). The tumor necrosis factor ligand-encoding gene *tnfsf4* and the glycine *N*-methyltransferase-encoding gene *gnmt* were upregulated in LPS-tolerant macrophages in the presence of carboplatin. Significantly upregulated pathways with an FDR < 0.05 included p53 pathway. Conversely, among the downregulated genes, *sox5,* encoding a transcription factor, was downregulated 18-fold. Hedgehog, E2F, mitotic spindle and G2/M checkpoint hallmark gene sets were statistically downregulated (FDR < 0.5, Fig. 4C). These results indicated that carboplatin treatment severely influenced cell cycle-associated genes.

**Table 2:** Lists of DEGs in LPS-tolerant macrophages in the presence of carboplatin, |log2FC| > 2, *p* < 0.25.

ENSEMBL ID	Gene name	Gene function	logFC	Adj. *p*-val
ENSMUSG00000025993	*Slc40a1*	Solute carrier family 40 (iron-regulated transporter)	3.41	0.0765
ENSMUSG00000019590	*Cyb561*	Cytochrome *b*-561	3.40	0.0959
ENSMUSG00000026700	*Tnfsf4*	Tumor necrosis factor (ligand) superfamily	3.38	0.1660
ENSMUSG00000002769	*Gnmt*	Glycine *N*-methyltransferase	3.28	0.1603
	*D830031N03Rik*	RIKEN cDNA D830031N03 gene	3.10	0.1614
ENSMUSG00000038569	*Rad9b*	RAD9 checkpoint clamp component B	2.89	0.0947
ENSMUSG00000025887	*Casp12*	Caspase 12	2.81	0.1316
ENSMUSG00000051984	*Sec31b*	Sec31 homolog B (S. cerevisiae)	2.76	0.2181
ENSMUSG00000021903	*Galnt15*	Polypeptide *N*-acetylgalactosaminyltransferase 14	2.33	0.2104
ENSMUSG00000021815	*Mss51*	MSS51 mitochondrial translational activator	2.14	0.1858

ENSEMBL ID	Gene name	Gene function	logFC	Adj. *p*-val
ENSMUSG00000037196	*Pacrg*	PARK2 coregulated-like	− 4.54	0.0224
ENSMUSG00000041540	*Sox5*	Transcription factor SOX-5	− 4.20	0.0680
ENSMUSG00000063873	*Slc24a3*	Sodium/potassium/calcium exchanger	− 4.19	0.1311
ENSMUSG00000001403	*Ube2c*	Phospholipase C	− 3.81	0.0765
ENSMUSG00000029516	*Cit*	Ubiquitin-conjugating enzyme E2C	− 3.55	0.2159
ENSMUSG00000027316	*Gfra4*	Glial cell line derived neurotrophic factor family receptor alpha 4	− 3.47	0.1548
ENSMUSG00000051177	*Plcb1*	Phospholipase C, beta 1	− 3.44	0.1309
ENSMUSG00000023826	*Prkn*	Parkin RBR E3 ubiquitin protein ligase	− 3.42	0.1209
ENSMUSG00000004668	*Abca13*	ATP-binding cassette, subfamily A (ABC1)	− 3.33	0.1504
ENSMUSG00000019768	*Esr1*	Estrogen receptor 1 (alpha)	− 3.32	0.0899
ENSMUSG00000060429	*Sntb1*	Syntrophin, basic 1	− 3.24	0.0780
ENSMUSG00000039578	*Ccser1*	Coiled-coil serine rich 1	− 3.24	0.1345
ENSMUSG00000019996	*Map7*	Microtubule-associated protein 7	− 3.14	0.1843
ENSMUSG00000031129	*Slc9a9*	Solute carrier family 9 (sodium/hydrogen exchanger)	− 3.13	0.1858
ENSMUSG00000026768	*Itga8*	Integrin alpha 8	− 3.10	0.1474
ENSMUSG00000050965	*Prkca*	Protein kinase C, alpha	− 3.07	0.1345
ENSMUSG00000056899	*Immp2l*	IMP2 inner mitochondrial membrane peptidase-like (*S. cerevisiae*)	− 3.05	0.1209
ENSMUSG00000063568	*Jazf1*	JAZF zinc finger 1	− 3.02	0.1953
ENSMUSG00000078922	*Tgtp1*	T cell specific GTPase 1	− 2.93	0.1311
ENSMUSG00000059060	*Rad51b*	RAD51 paralog B	− 2.84	0.1396
ENSMUSG00000021097	*Clmn*	Calmin	− 2.78	0.0935
ENSMUSG00000036777	*Anln*	Anillin	− 2.74	0.1646
ENSMUSG00000038372	*Gmds*	GDP-mannose 4,6 dehydratase	− 2.61	0.0526
ENSMUSG00000035441	*Myo1d*	Myosin ID	− 2.60	0.2417
ENSMUSG00000063458	*Lrmda*	Leucine rich melanocyte differentiation associated	− 2.56	0.0959
ENSMUSG00000049744	*Arhgap15*	Rho GTPase-activating protein	− 2.52	0.0302
ENSMUSG00000045667	*Smtnl2*	Smoothelin-like 2	− 2.48	0.1107
ENSMUSG00000020598	*Nrcam*	Neuronal cell adhesion molecule	− 2.46	0.2104
ENSMUSG00000054733	*Msra*	Methionine sulfoxide reductase A	− 2.39	0.0269
ENSMUSG00000031274	*Col4a5*	Collagen, type IV, alpha 5	− 2.38	0.1660
ENSMUSG00000022021	*Diaph3*	Protein diaphanous homolog 3	− 2.37	0.2453
ENSMUSG00000039109	*F13a1*	Coagulation factor XIII A chain	− 2.28	0.1970
ENSMUSG00000047921	*Trappc9*	Trafficking protein particle complex subunit 9	− 2.28	0.0008
ENSMUSG00000030867	*Plk1*	Serine/threonine-protein kinase PLK1 (polo like kinase 1)	− 2.25	0.2104
ENSMUSG00000052928	*Ctif*	Cliff drop aversion	− 2.17	0.1311
ENSMUSG00000035919	*Bbs9*	Protein PTHB1	− 2.12	0.0254
ENSMUSG00000056602	*Fry*	FRY microtubule binding protein	− 2.12	0.1558
ENSMUSG00000061533	*Cep128*	Centrosomal protein of 128 kDa	− 2.07	0.1162
ENSMUSG00000036278	*Macrod1*	ADP-ribose glycohydrolase MACROD1	− 2.07	0.0165
ENSMUSG00000028080	*Lrba*	Lipopolysaccharide-responsive and beige-like anchor protein	− 2.01	0.0165

Because carboplatin downregulated genes involved in cell cycle transition, we next tested whether carboplatin interfered with cell cycle progression upon LPS stimulation using bromodeoxyuridine (BrdU) analysis. LPS priming alone significantly reduced BrdU uptake, while carboplatin treatment did not change the level of BrdU incorporation (Fig. 4D). On the other hand, as predicted from the transcriptomic analysis, a significant reduction in BrdU uptake was observed in carboplatin-treated LPS-tolerant macrophages. Therefore, we confirmed that carboplatin treatment during LPS priming led to reduced cell cycle progression in LPS-tolerant BMDMs.

### Effect of carboplatin on global histone modifications

**Figure 5:**
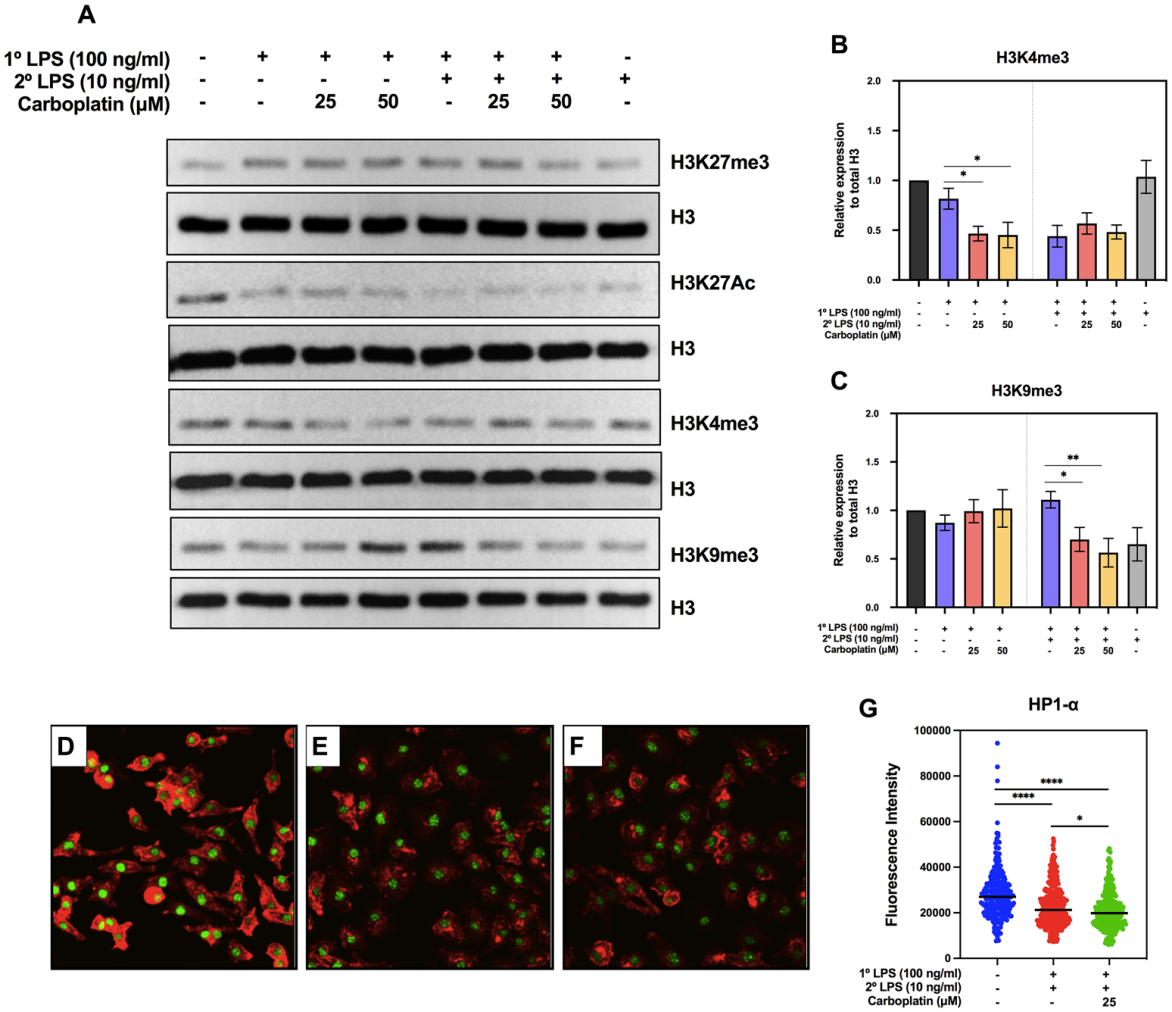
Effects of carboplatin on global histone modification profiles and the level of HP1-α. (**A**) Histone modification profiles of LPS-primed and LPS-tolerant macrophages with or without carboplatin are shown. Methylation profiles of H3K27me3, H3K27Ac, H3K4me3, and H3K9me3 expression were analyzed by western blotting. Total H3 was used as control. The data shown are representative blots of replicates (n ≥ 3). (**B**,**C**) The relative band intensities of H3K4me3 and H3K9me3 were measured by ImageJ analysis and normalized to the total H3 levels. *, ** indicate *p* < 0.05, *p* < 0.01, respectively, using one-way ANOVA. (**D**–**G**) Immunofluorescence staining of HP1-α is shown. BMDMs were stained with rabbit anti-HP1-α antibody and detected using anti-rabbit IgG antibody conjugated to Alexa Fluor^®^ 488 (green), followed by staining with phalloidin (red). (**D**) Untreated BMDMs, (**E**) LPS-tolerant BMDMs without carboplatin and (**F**) with carboplatin. The images were acquired using a confocal microscope. (**G**) Fluorescence intensity was measured and calculated based on the corrected total cell fluorescence (CTCF) method. The results shown represent the means ± SD, n = 3 and p < 0.05.

It was previously reported that carboplatin altered histone modifications such as histone H3 lysine 4 trimethylation (H3K4me3) and histone H3 lysine 9 acetylation (H3K9) at reactivated genes in cancer cells^13^. Because LPS tolerance is partially regulated by histone modification, we therefore investigated global histone profiles, including repressive marks (H3K27me3 and H3K9me3) and active marks (H3K4me3 and H3K27Ac), during LPS priming and LPS re-exposure with or without carboplatin. Cell lysates were harvested for western blot analyses after 24 h of LPS priming or following 3 h of secondary LPS stimulation, with or without carboplatin (25 or 50 µM). The levels of H3K27me3 were maintained at relatively constant levels in all conditions (Fig. 5A, Supplementary Figs. S7, S8). The level of H3K27Ac decreased with LPS priming and remained at a low level during LPS re-exposure regardless of carboplatin treatment. However, significant decreased H3K4me3 by carboplatin treatment during priming was observed but the difference was not found in LPS-tolerant macrophages (Fig. 5A,B). Moreover, a significant reduction in H3K9me3 level was detected in carboplatin-treated LPS-tolerant macrophages (Fig. 5A,C; Supplementary Figs. S7, S8). These results suggest that carboplatin may interfere with global histone modifications.

### Carboplatin decreased HP1-α in LPS-tolerant macrophages

Because we observed increased TNF-α/IL-6 in carboplatin-treated macrophages with increased global H3K9me3 levels, we asked whether carboplatin affects the levels of HP1-α, which is associated with H3K9me3-mediated gene silencing. We performed immunofluorescence staining for HP1-α with or without carboplatin in LPS-tolerant BMDMs. HP1-α was exclusively detected in the nuclei under all conditions. The level of HP1-α fluorescence intensity in LPS-tolerant macrophages was significantly decreased compared to the control (Fig. 5D–F and G). More importantly, the immunofluorescence intensity was significantly decreased under carboplatin treatment in LPS-tolerant macrophages (Fig. 5G).

### Changes in the transcripts of H3K9 modifying enzymes induced by carboplatin

**Figure 6:**
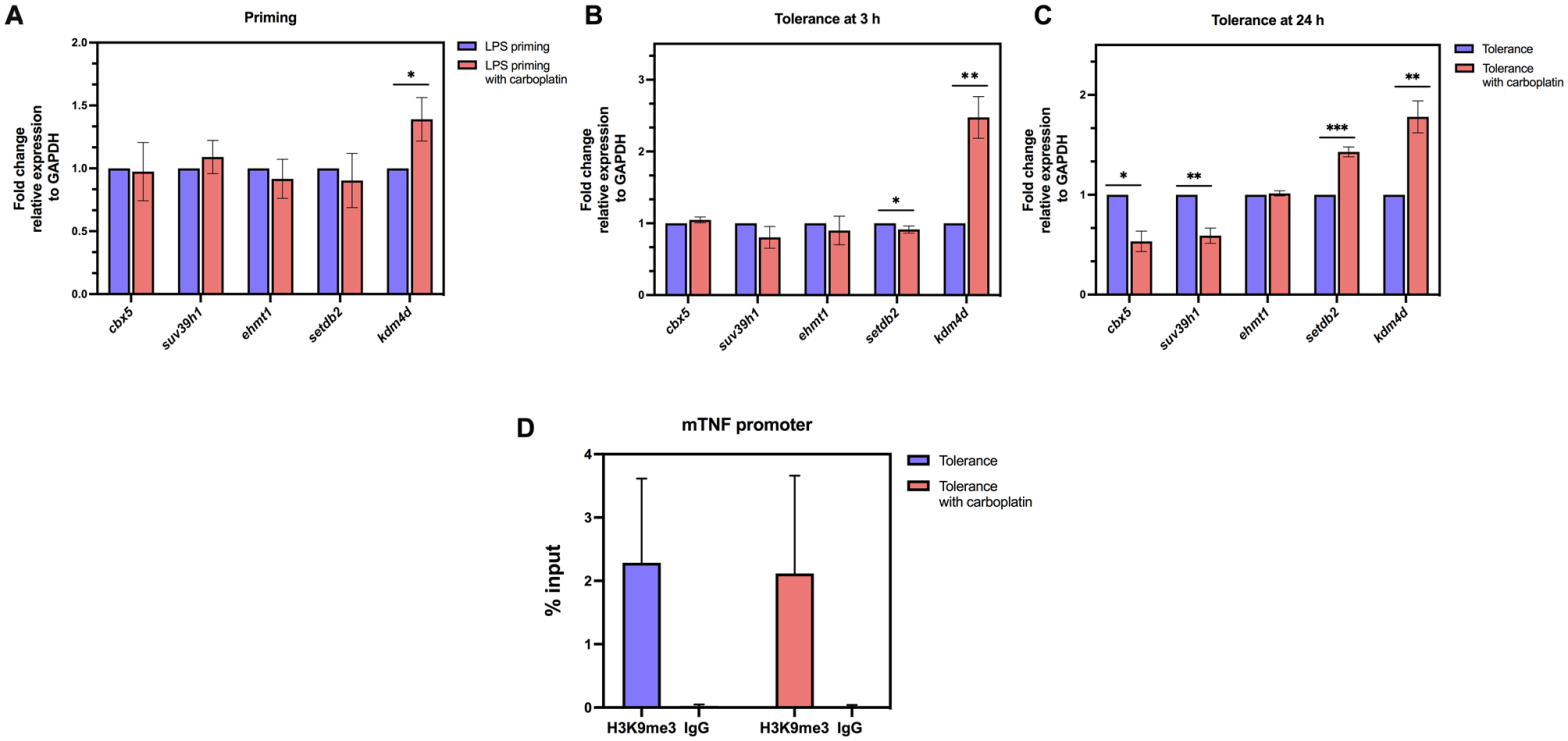
Effects of carboplatin on the expression of genes encoding H3K9-modifying enzymes. (**A**,**B**) BMDMs were primed and challenged with LPS as described. The relative expression of the following genes encoding H3K9-modifying enzymes was quantified by RT-qPCR: the HP1-α encoding gene *cbx5;* methyltransferase enzyme coding genes *suv39h1, ehmt1,* and *setdb2;* and the demethylase enzyme encoding gene *kdm4d*. (**A**) LPS-primed BMDMs and (**B**,**C**) LPS-tolerant BMDMs at 3 h or 24 h. Transcript levels are expressed relative to the untreated control after normalization to the housekeeping gene *gapdh.* Experiments were performed in at least 2 biological replicates. *, **, ***, **** indicate *p* < 0.05, *p* < 0.01, *p* < 0.001, *p* < 0.0001, respectively, using one-way ANOVA. (**D**) ChIP-qPCR of H3K9me3-associated DNA spanning the *tnf-α* promoter. BMDMs were treated as indicated with or without carboplatin. Cells were fixed and prepared according to the SimpleChIP^®^ Enzymatic Chromatin IP kit. The promoter enrichment quantification was normalized to a 2% input. Rabbit anti-IgG antibody was used as negative control. The assay was conducted in 3 biological replicates.

Based on the effects of carboplatin observed thus far, we next asked whether H3K9-modifying enzymes were altered by carboplatin treatment. Enzymes that mediate H3K9 modification include lysine methyltransferase enzymes encoded by *suv39h1*, *ehmt1*, and *setdb2*; the demethylase enzyme *kdm4d*; and the HP1-α encoding gene, *cbx5*. During the LPS priming period, we did not find significant differences in the expression of any of the methyltransferase enzyme genes or the HP1-α-encoding gene *cbx5*. However, the demethylase enzyme *kdm4d* was highly upregulated in carboplatin-treated macrophages (Fig. 6A). Upon 3 h incubation with secondary LPS (the procedure scheme shown in Fig. 1A), we found significantly lower *setdb2* transcripts (approximately 9% reduction) and higher *kdm4d* mRNA levels in carboplatin-treated cells than in control cells (Fig. 6B). We then further investigated the expression of these H3K9-modifying enzyme encoded genes in which the re-exposure time was extended to 24 h. As expected, the level of *cbx5* and *suv39h1* were significantly decreased in carboplatin treated cells than control LPS tolerant macrophage. The *kdm4d* remained up-regulated upon carboplatin treatment for 24 h. To our surprise, the treatment with carboplatin for 24 significantly increased the level of *setdb2* comparing to the LPS tolerant macrophages without carboplatin (Fig. 6C). Taken together, these results suggested that carboplatin treatment during LPS priming may alter H3K9me3 modifications by enhancing the expression of the demethylase-encoding gene *kdm4d* and reducing the expression of the methyltransferase-encoding gene *setdb2* and *suv39h1* with different temporal kinetics.

Finally, we aimed to determine whether H3K9me3 modification influences the production of TNF-α in carboplatin-treated tolerant macrophages by performing ChIP-PCR at the cis-regulatory regions of the *Tnf* gene. Unexpectedly, we found no differences in the level of H3K9me3 associated with the *Tnf* promoter in the control and carboplatin-treated macrophages (Fig. 6D).

## Discussion

In this study, we reported the effect of a platinum-containing anticancer chemotherapy drug, carboplatin, on the response of macrophages to LPS and LPS tolerance. Carboplatin treatment increased IL-6 production during LPS priming and rescued the repressed production of TNF-α and IL-6 during LPS tolerance. This effect of carboplatin did not involve its impact on cell viability or the immediate signaling pathways downstream of TLR4. Transcriptomic analysis by GSEA revealed that carboplatin treatment increased the hallmark gene sets associated with the p53 pathway and decreased those associated with the glycolysis pathway. Histone modifications were altered, which indicated the involvement of epigenetic modifications. Because carboplatin is used to treat some solid tumors, the impact on macrophage responses highlights its potential systemic influences on immune responses.

Transcriptomic approaches pointed out the likely mechanism by which this drug modified the macrophage response to LPS. Carboplatin and related chemotherapeutic drugs are known to inhibit DNA/RNA synthesis by forming adducts with DNA and disturbing its structure, which eventually leads to cell death^23^. We noticed that *smarcd3* was upregulated upon carboplatin treatment in LPS-primed macrophages (Table 1). This gene encodes an actin-dependent regulator of chromatin and is involved in the SWI/SNF (SWItch/Sucrose Non-Fermentable) complex^24^. The SWI/SNF complex changes chromatin by repositioning nucleosomes, emitting nucleosome octamers or expelling histone dimers^25^. A previous study revealed low proliferation rates in SMARCD3-depleted cells, reflecting a failure of cell cycle progression^26^. Moreover, p21 accumulated in SMARCD3-depleted cells, but the cell cycle was not halted, resulting in the accumulation of unrepaired DNA damage. Therefore, based on our data, increase in the expression of *smarcd3* by carboplatin treatment potentially results in a remodeling of chromatin and a subsequent alteration in gene expression in response to LPS.

Remarkably, the hallmark p53 pathway was one of the positively regulated pathways in carboplatin treatment in both the LPS priming and tolerance states. P53 is crucial in oncogenesis prevention, but its roles in macrophage function and inflammation are not well understood. A previous study reported that when BMDMs are polarized to the M2 subtype (alternatively activated macrophages), they have increased endogenous p53 activity^27^. M2-polarized macrophages were found to be necessary for the development of tolerance to LPS^28^. More importantly, tolerance to LPS was associated with decreased p53 activity^27^. Conversely, p53 activation by exposure to Nutlin-3a, a small-molecule MDM2 inhibitor that destabilizes the MDM2-p53 complex, reduces tolerance development.

Another hallmark pathway related to glycolysis was also negatively regulated. LPS treatment leads to glycolytic reprogramming of murine macrophages, which is required for cell survival and anti-inflammatory cytokine production^29^. In addition, among the small subset of upregulated genes, *nr4a2* (*nurr1*), a gene encoding orphan nuclear hormone receptor, was highly upregulated by carboplatin treatment during LPS priming. NR4A2 promotes alternative polarization of macrophages and protects mice against sepsis^30^. Therefore, it is possible that carboplatin treatment may result in decreased glycolytic reprogramming that affects the LPS response and leads to the switch to an M2-like phenotype.

Because carboplatin treatment can lead to cell death, we used the MTT and BrdU incorporation assays to address this in our model. No obvious cell toxicity was observed, at least for the duration of our experiments, but a significant decrease in BrdU uptake was found. Macrophages are believed to be terminally differentiated and do not actively undergo cell division. Recent evidence indicates that macrophages maintain homeostatic proliferation in the presence of mitogens such as CSF-1 in a Myc-dependent manner. Proinflammatory stimuli, however, counteract this proliferation by suppressing Myc expression^31^. This result is consistent with our BrdU assay, where unstimulated BMDMs took up more BrdU than LPS-primed cells. More importantly, upon carboplatin treatment, LPS-tolerant macrophages halted BrdU incorporation. This is consistent with our data reporting that the G2/M checkpoint was negatively regulated.

Recent evidence has shown that the activation of p53 regulates several H3K9 methylation enzymes^32^. p53 directly induces the H3K9 demethylase, Jumonji domain 2 family demethylase (JMJD2b), via promoter binding and indirectly downregulate the expression of H3K9 methyltransferase SUV39H1^32^. In addition, KDM4D (also known as JMJD2D), an H3K9 demethylase, forms a complex with p53 or interacts with the p53 DNA binding domain, where its catalytic activity is required to stimulate p53-dependent transcription^33^. In agreement with our data, the H3K9 methyltransferase-encoding gene *setdb2* was downregulated, while *kdm4d*, a gene encoding the H3K9 demethylase, was more highly expressed in the presence of carboplatin in LPS-tolerant BMDMs. This modification of H3K9 may be partially related to the positive regulation by p53. SETDB2-specific depletion in macrophages impaired the transition from inflammatory to reparative macrophages. Trimethylation of H3 by SETDB2 was reported to be at the NF-κB binding sites in the promoters of inflammatory cytokine genes and subsequently repressed transcription^34^. Based on our results, the s*etdb2* expression is down-regulated at the early time but increased after 24 h while the *suv39h1* was dramatically repressed. This result may indicate that the dynamic changes in enzyme levels that can modulate H3K9, together with the decreased *cbx5*, are responsible for reduced H3K9me3. Taken together, we propose that carboplatin treatment rescues the production of proinflammatory cytokines in LPS-tolerant macrophages by inducing dynamic changes in histone lysine methyltransferases KDM4D and SUV39H1 (only at late timepoint) and SETDB2 (only at early time point), thereby modifying the level of H3K9me3. Taken together, we propose that carboplatin treatment rescues the production of proinflammatory cytokines in LPS-tolerant macrophages by inducing the KDM4D and reducing SETDB2, SUV39H1, thereby modifying H3K9me3.

Heterochromatin protein 1 (HP1) is a transcriptional corepressor associated with heterochromatin formation. It recognizes and binds to H3K9me3^35^. However, the mechanisms underlying HP1-induced heterochromatin are not well defined. This self-maintaining mechanism of HP1-bound H3K9me3 amplifies the silencing effect of HP1 along areas in its vicinity. Previous evidence has shown that the reduced HP1-α levels in VSMCs was consistent with lower levels of SUV39H1^36^. Additionally, carboplatin has been indicated to potentially inhibit HP1-α expression in YB5 cells^13^. Therefore, we suggest that carboplatin drove the reduction in HP1-α, leading to increased instability of HP1-bound H3K9me3.

Based on our observations, global H3K4me3 and H3K27Ac were decreased, while H3K9me3 increased upon LPS re-exposure compared to primary LPS stimulation (Fig. 5A), and carboplatin treatment slightly reversed this trend. Carboplatin appears to affect a specific set of genes, termed tolerizeable genes, by increasing their expression while leaving nontolerizeable genes unaltered (Supplementary Fig. S9). H3K9me3 can be found at poised enhancer sequences^37^. It has also been shown that before stimulation, bone marrow-derived DCs have low levels of H3K9me3 at their *Mdc* and *Il12b* promoter loci, while H3K9me3 is enriched in their enhancer regions^38^. Moreover, it has been reported that KDM4D (JMJD2D) demethylates H3K9me3 around enhancers upon stimulation^38^. Since we observed global H3K9me3 by western blot, it is possible that H3K9me3 marks were not present in the promotor of *tnf-α* but on its enhancer.

The limitation of our study is the unexplored effect of carboplatin in vivo where LPS tolerance is observed such as in sepsis. Sepsis is a systemic inflammatory condition when massive cytokine storms in response to infection is followed by depressed immune response called immune paralysis^39^. Animal models such as two-hit cecal ligation and puncture (CLP), may be used to test whether carboplatin can rescue immune paralysis phenotypes^40^. One caveat here is that carboplatin treatment may have hematopoietic toxicity which results in reduced immune cell output, such as neutropenia^41^. Therefore, systemic application of carboplatin may negatively affect viability hematopoietic cells which may complicate the interpretation of the outcomes.

Based on our findings, we propose a mechanism by which carboplatin affects LPS tolerance, as shown in Supplementary Fig. S10. Carboplatin treatment increases p53-related signaling cascades, leading to disrupted cell cycle progression. In addition, the treatment leads to drastic changes in histone modifying enzymes that drive transcription of tolerizeable genes in LPS-tolerant macrophages. This finding may have implications for the side effects on innate immune memory of carboplatin treatment in cancer patients.

## Materials and methods

### Bone marrow-derived macrophages (BMDMs)

Female mice (C57BL/6; Nomura Siam International, Thailand) were used in this study. Mice were humanely sacrificed using inhalant anesthetic isoflurane overdose. BMDMs were harvested from the femurs and tibias by flushing. Bone marrow cells were cultured for 7 days in Dulbecco's modified Eagle's medium (DMEM, HyClone, Logan, UT, USA) supplemented with 10% (v/v) fetal bovine serum (Gibco, Grand Island, NY, USA), 1% (w/v) sodium pyruvate, 1% (w/v) HEPES, 100 U/ml pen/strep, 20% L929 cell conditioned media and 5% horse serum. On day 7, macrophages were resuspended at a density of 1 × 10^6^ cells/ml, and plated at 2 × 10^5^ cells were plated in culture plates and allowed to adhere for 16–18 h prior to use. All experimental procedures involving laboratory animals were approved by the Institutional Animal Care and Use Committee (IACUC) of the Faculty of Medicine, Chulalongkorn University (approval protocol No. 025/2562). All experiments were performed according to the guidelines issued by the IACUC.

### LPS and carboplatin treatments

BMDMs were pretreated with carboplatin (25 or 50 µM) (Selleckchem, Houston, TX USA) or vehicle control in BMDM differentiation media (BMM) for 1 h prior to *Salmonella* spp*.* LPS (Sigma Aldrich, St. Louis, MO, USA) stimulation (100 ng/ml) for 24 h. After 24 h incubation, media were removed, fresh media with LPS (10 ng/ml) was added, and the cells were cultured for 3 or 24 h as indicated^42^. Supernatants were collected for ELISAs. A schematic of the procedure is shown in Fig. 1A.

### Enzyme-linked immunosorbent assay (ELISA)

The amount of TNF-α and IL-6 was quantified in culture supernatants from BMDMs treated as indicated using mouse TNF-α ELISA and IL-6 ELISA kits (BioLegend, CA, USA) following the manufacturer’s instructions. Streptavidin HRP was used to detect bound antibodies, and TMB (Sigma Aldrich) was used as a substrate. The reaction was stopped with 1 M H_2_SO_4_. Absorbance at 450 and 620 nm was measured on a microplate reader (Thermo Fisher Scientific).

### MTT and BrdU incorporation assays

Cellular toxicity was determined using a tetrazolium dye 3-(4,5-dimethylthiazol-2-yl)-2,5-diphenyltetrazolium (MTT) assay (Thermo Fisher Scientific, Wilmington, DE, USA) according to the manufacturer's instructions. Briefly, BMDMs at 1 × 10^5^ cells/well were treated as indicated and incubated with 0.5 mg/ml MTT solution for 4 h at 37 °C. Formazan was dissolved in dimethyl sulfoxide (DMSO; Thermo Fisher Scientific) prior to measurement of absorbance at 540 nm by a microplate reader.

Cell proliferation was assessed by a BrdU Cell Proliferation Assay (Merck-Millipore, Germany) according to the manufacturer’s protocol. Briefly, approximately 2 × 10^5^ BMDMs were treated as described, followed by BrdU labeling for an additional 2 h. Cells were fixed and permeabilized before adding the anti-BrdU antibody. The reaction was quantified by a microplate reader at an absorbance of 450 nm.

### Reverse transcription and qPCR (RT-qPCR)

Total RNA was isolated using a Direct-zol RNA isolation kit (Zymo Research, CA, USA) and reverse transcribed using RevertAid reverse transcriptase (Thermo Fisher Scientific) following the manufacturer's protocol. Quantitative real-time PCR (qPCR) was carried out using iQ SYBR^®^ Green Supermix (Bio-Rad) and a CFX Connect Real-Time PCR Detection System (Bio-Rad). Relative expression was calculated by normalizing to *beta-actin* or *gapdh* as housekeeping genes. A list of primers used in this study is shown in Supplementary Table S3. The results were calculated and presented as relative quantifications using the 2^−∆∆ct^ method.

### Western blotting

BMDMs were treated as indicated. Whole-cell lysates were prepared in RIPA buffer and subjected to western blotting. The antibodies used were rabbit anti-HP1-α, rabbit anti-H3K4me3, rabbit anti-H3K9me3, rabbit anti-H3K27me3, rabbit anti-H3K27Ac, rabbit anti-H, rabbit anti-NF-B, rabbit anti-ERK, rabbit anti-MAPK, rabbit anti-p-SAPK-JNK, rabbit anti-NF-κB, rabbit anti-ERK, rabbit anti-MAPK, rabbit anti-SAPK-JNK, mouse anti-actin, HRP-conjugated donkey anti-rabbit IgG and HRP-conjugated sheep anti-mouse IgG (all purchased from Cell Signaling Technology, Danvers, MA, USA). The signals were detected by chemiluminescence. The protein bands intensities were quantitated using ImageJ Gel Analysis program. The modified proteins were normalized to their loading controls (total forms) and relatively normalized to those of the unstimulated cells.

### Immunofluorescence staining

BMDMs were cultured at 1 × 10^5^ cells/well in 8-well chamber slides (Thermo Fisher Scientific). Cells were treated as indicated and fixed with 4% paraformaldehyde (Sigma-Aldrich), followed by permeabilization with 0.1% Triton-X 100 (Amersham Biosciences, Little Chalfont, UK). Cells were stained with rabbit anti-HP1-α antibody (Cell Signaling Technology) overnight, followed by staining with phalloidin according to the manufacturer’s instructions (Thermo Fisher Scientific). Fluorescent dye-conjugated secondary antibodies, including anti-rabbit IgG antibody conjugated to Alexa Fluor^®^ 488 (Cell Signaling Technology), were used for detection. Images were acquired with a confocal microscope (Olympus, Tokyo, Japan). Fluorescence intensity was quantified based on the corrected total cell fluorescence (CTCF) method with an open source image processing pipeline using python^43^.

### Library preparation and RNA-sequencing (RNA-Seq)

Total RNA was extracted using a Direct-zol RNA isolation kit (Zymo Research, CA, USA) following the manufacturer's protocol. Briefly, all experiments were conducted with three biological replicates. Total RNA concentration was quantified using a Nanodrop spectrophotometer (Thermo Fisher Scientific). RNA quality was assessed using the 2100 Bioanalyzer (Agilent Technology, CA, USA). RNA integrity number (RIN) scores greater than 7.0 were considered acceptable for further library preparation and RNA sequencing. cDNA libraries were prepared using a TruSeq stranded mRNA library preparation kit (Illumina, San Diego, CA, USA). The quantity and quality of the libraries were determined using a 2100 Bioanalyzer. Sequencing was carried out on a HiSeq (2 × 150 bp paired-end reads) at the Omics Sciences and Bioinformatics Center, Chulalongkorn University, Bangkok, Thailand. This study is reported in accordance with the ARRIVE guidelines.

### RNA-Seq analysis

Sequencing reads were mapped against the *Mus musculus* reference genome GRCm39. Reads were mapped and aligned with HISAT2. Reads were counted by HTSeq-count^44^. Subsequently, DEGs were compared and analyzed in R version 4.0.3 using the package DESeq2^45^. The analyses were conducted from triplicate samples. Genes were considered differentially expressed when the log_2_ fold change was < − 2 or > 2 (representing down- or upregulation, respectively) and the adjusted *p*-value was < 0.05 or 0.25, depending on conditions.

### Gene set enrichment analysis (GSEA)

Gene expression levels were converted to fold changes in gene expression over the median expression levels of the same genes on the population level. These genes were preranked in descending order and subjected to gene set enrichment analysis (GSEA)^46^ using the ‘ClusterProfiler’ R package^47^. Significantly enriched hallmark gene sets were determined using the following criteria: Benjamini–Hochberg (BH)-adjusted p-value < 0.05 and FDR q-value < 0.05.

### ChIP-qPCR

Approximately 1 × 10^7^ BMDMs were prepared and activated as described.

The SimpleChIP^®^ Enzymatic Chromatin IP Kit (Cell Signaling Technology) was used according to the manufacturer's instructions. Samples were subjected to immunoprecipitation using either rabbit anti-H3K9me3 antibody or a control IgG antibody. Fragmented DNAs were isolated from histones by Proteinase K and purified using spin columns (both from Cell Signaling Technology). DNA was used as a template for qPCR using the indicated primer sets spanning the *tnf-*α promoter (Supplementary Table S3). Fold enrichments were normalized and calculated based on the total amount of 2% input and presented as a relative quantifications using the 2^−∆∆ct^ method.

### Statistical analyses

Significant differences between two independent samples were determined by an unpaired t-test. One-way ANOVA with Dunnett’s post hoc test was used to identify significant differences among samples in one group. *p* < 0.05 was considered statistically significant.

## Supplementary Information

Supplementary Figures.

Supplementary Tables.

## Data Availability

The datasets generated or analyzed for this study were deposited in a public database and can be found under the GEO accession number GSE179974.
