# Supplementary material for: The chemotherapeutic drug carboplatin affects macrophage responses to LPS and LPS tolerance via epigenetic modifications
Source: Sci Rep. 2021 Nov 3;11:21574. doi: 10.1038/s41598-021-00955-7 (PMC8566489; doi:10.1038/s41598-021-00955-7)
Supplement: Supplementary file 1 — Supplementary Figures. [file 41598_2021_955_MOESM1_ESM.docx]

**Carboplatin Interferes with LPS-induced Tolerance in Macrophages via Epigenetic Modifications**

Boonmee et al.

**Supplementary Figures**


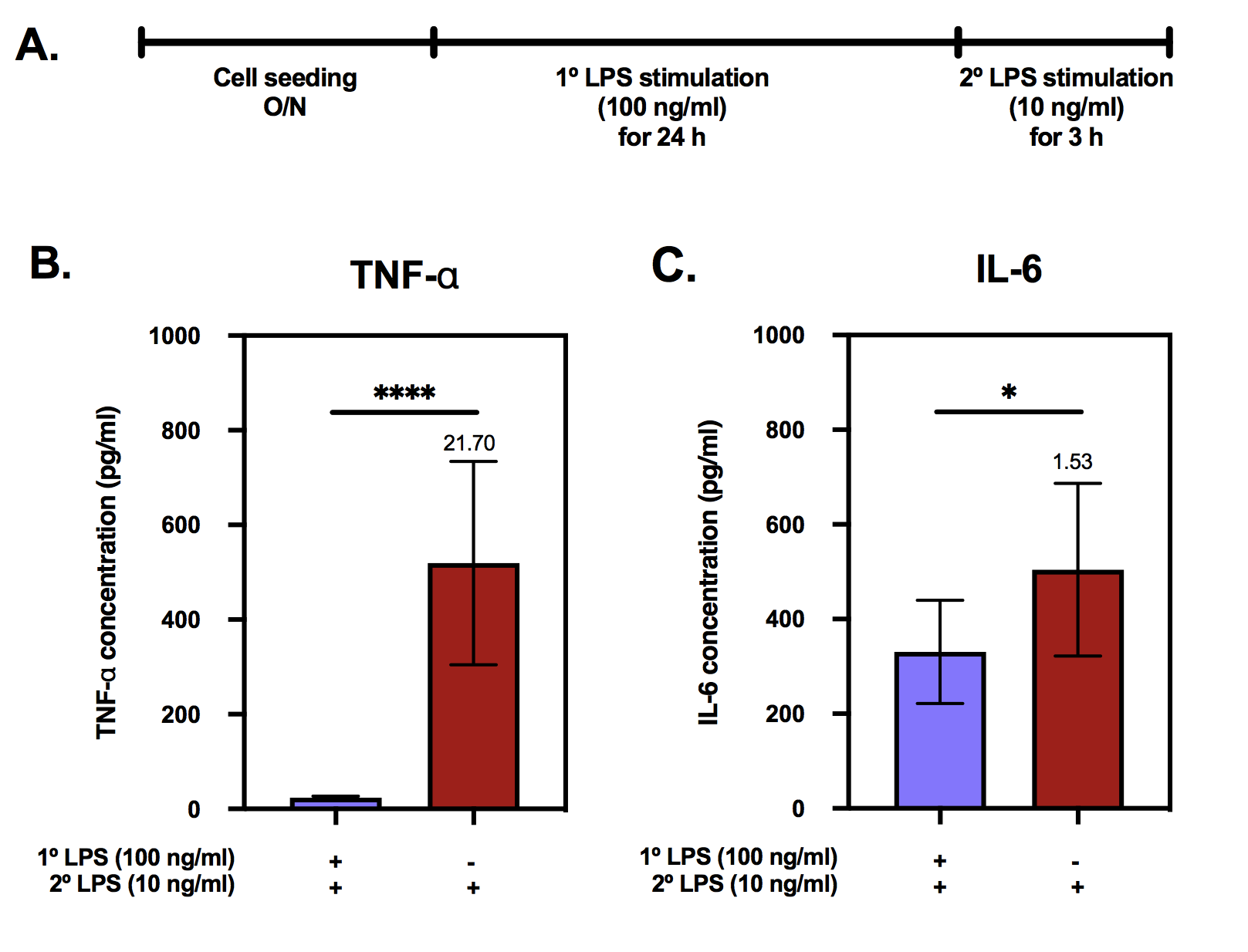


**Supplementary Fig. S1.** **TNF-α and IL-6 ELISA of LPS-stimulated macrophages.**

**(A)** Schematic protocol for treatment of LPS-tolerant macrophages. **(B, C)** Cytokine production (**(B)** TNF-α **(C)** IL-6)) from LPS-tolerant macrophages and the single dose LPS (10 ng/ml) control. Data are representative of at least 3 independent experiments. **, ****; *p* < 0.05, *p* < 0.0001, respectively, using unpaired t-test. The numbers above the bar indicate fold differences when compared with the non-treated cells.


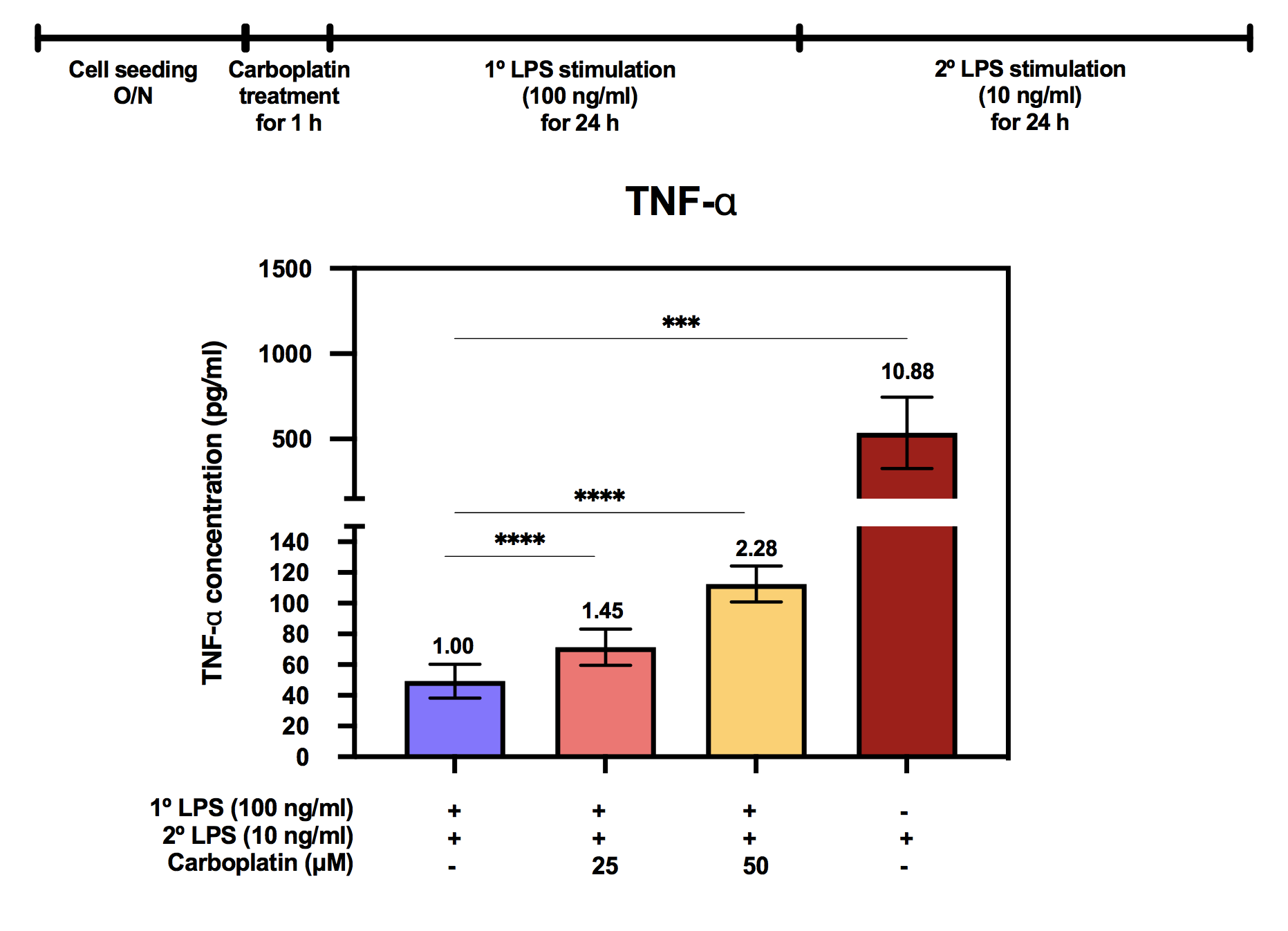


**Supplementary Fig. S2.** **Production of** **TNF α in LPS-tolerant macrophages with or without carboplatin after 24 hr of incubation.**

TNF-α level in carboplatin and non-treated LPS-tolerant macrophages after 24 h of incubation in the secondary LPS stimulation. Data are representative of at least 3 independent experiments. ***, ****; *p* < 0.001, *p* < 0.0001, respectively, using One-way ANOVA. The numbers above the bar indicate fold differences when compared with the non-treated cells.


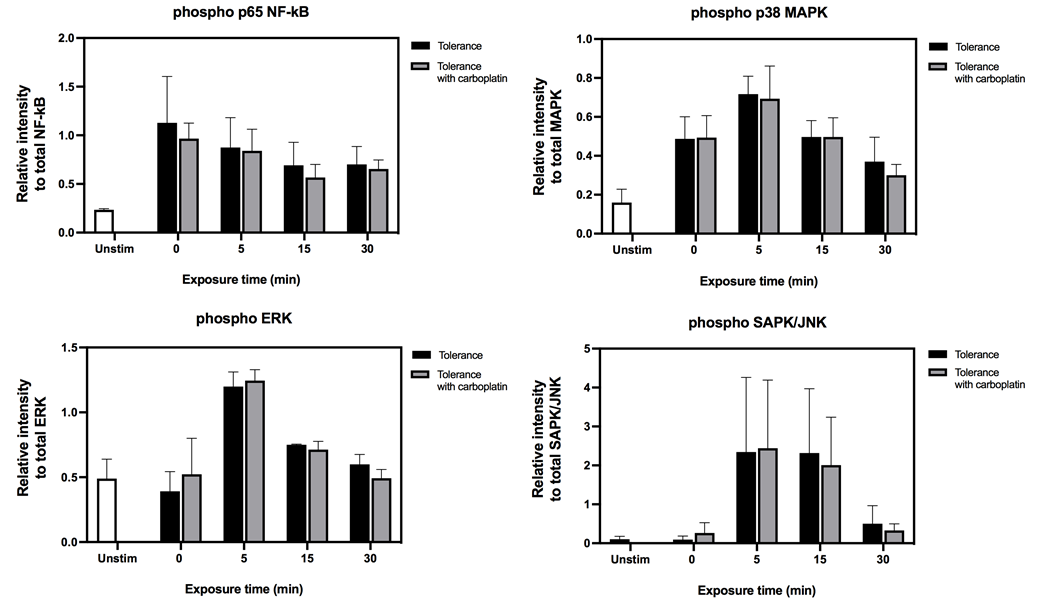


**Supplementary Fig. S3.** **Effects of carboplatin on the TLR4 signaling pathway.**

Downstream TLR-4 signaling pathway activity in LPS-primed and LPS-tolerant macrophages with or without carboplatin was detected by Western blot as described in Figure 2. The relative band intensities of NF-kB , ERK, p38, SAPK/JNK were measured by ImageJ analysis and normalized to the total level of each protein.

**
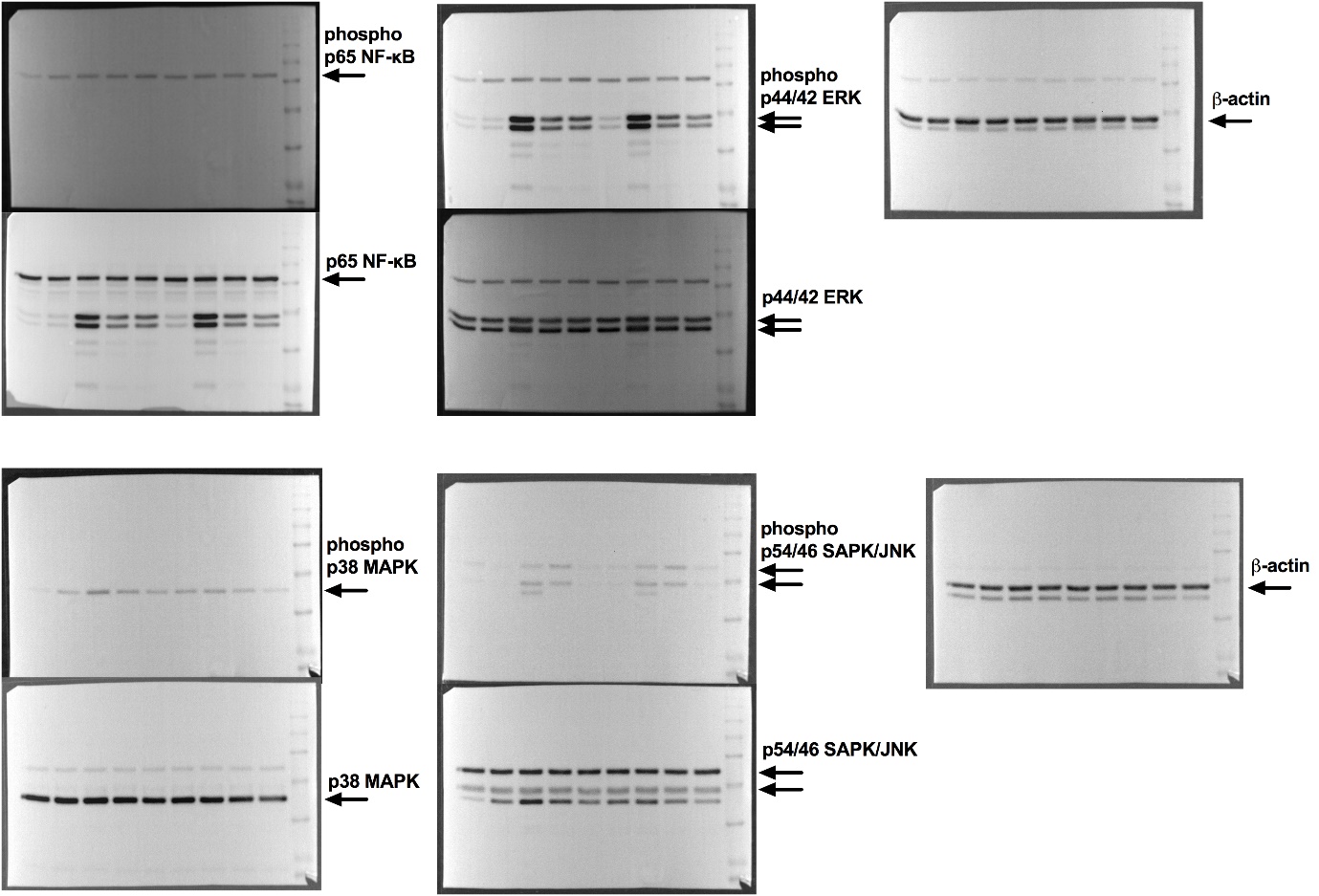
**

**Supplementary Fig. S4. Representative Western blots to investigate the effects of carboplatin on the TLR4 signaling pathway.**

Representative of unprocessed Western blots to analyze signaling pathways down stream of TLR4 as described in Figure 2 are shown.


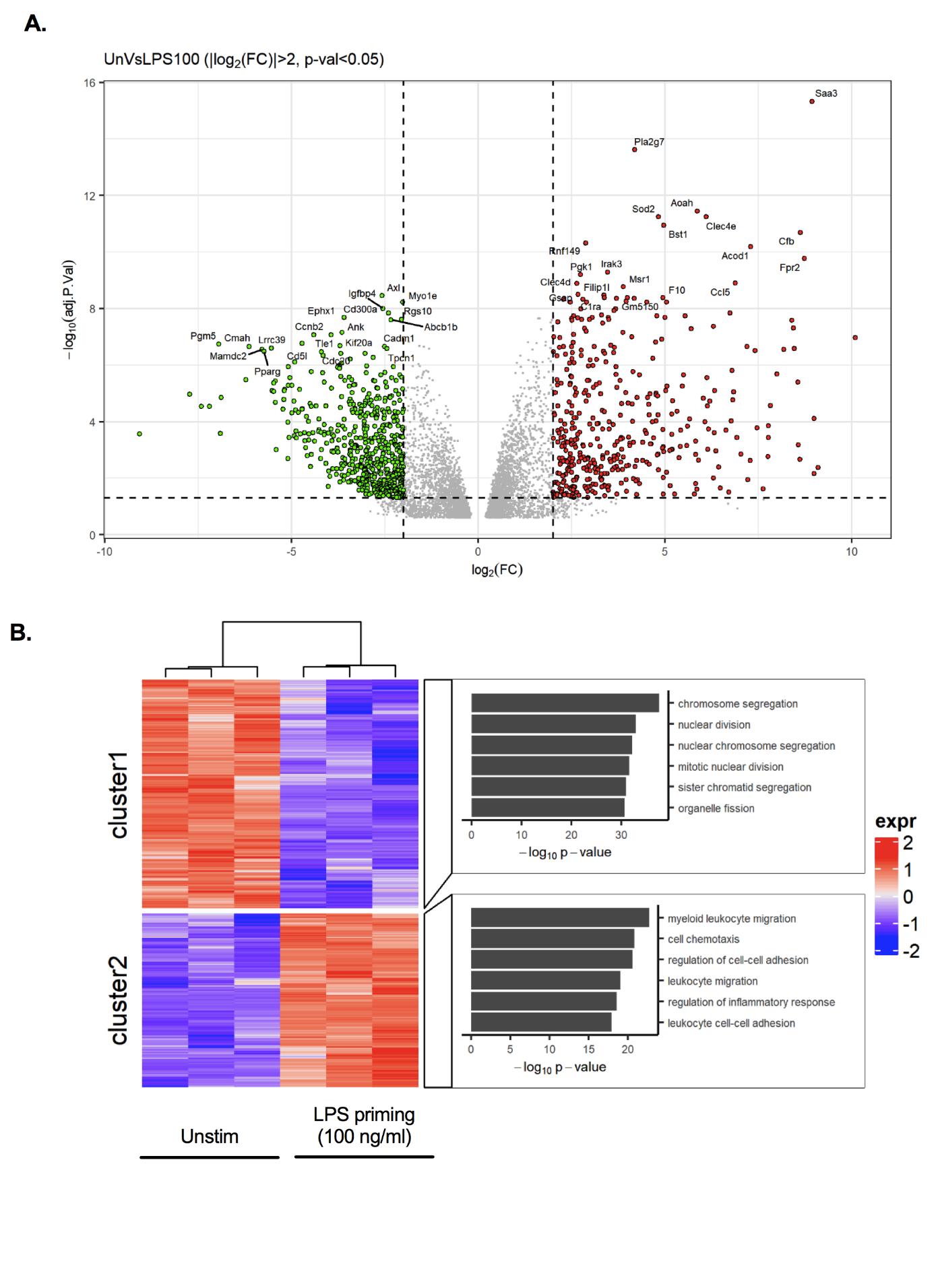


**Supplementary Fig. S5.** **Transcriptomic profiles of LPS-primed macrophages.**

**(A)** Volcano plot depicting DEGs of LPS-primed macrophages, compared with unstimulated cells. |log2FC| > 2, *p* < 0.05. **(B)** Heatmap and the GO-terms associated with DEGs.


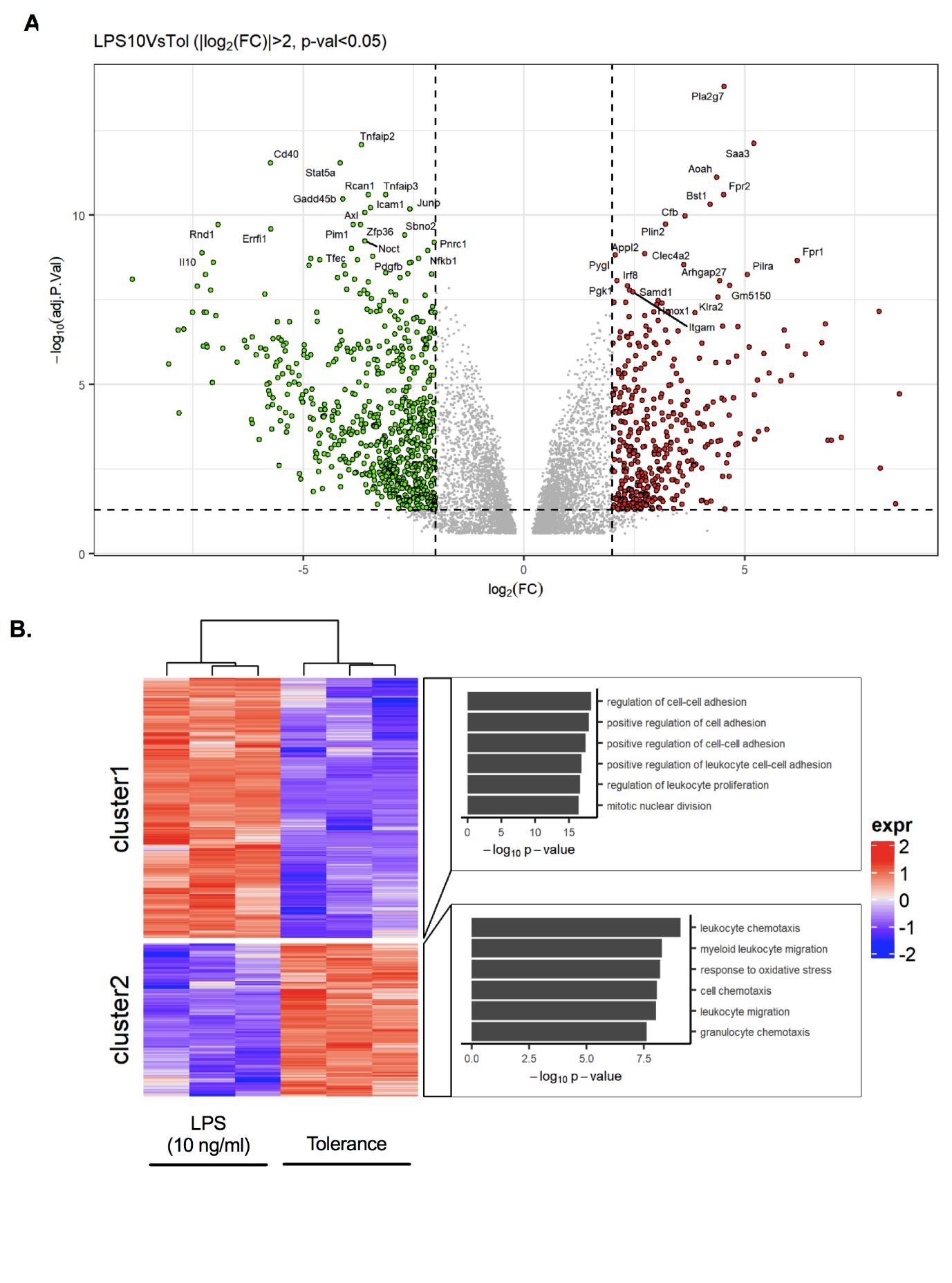


**Supplementary Fig. S6.** **Transcriptomic of LPS-tolerant macrophages.**

**(A)** Volcano plot depicting DEGs of LPS-tolerant macrophages, compared with LPS stimulated control. |log2FC| > 2, *p* < 0.05. **(B)** Heatmap and the GO-terms associated with DEGs.


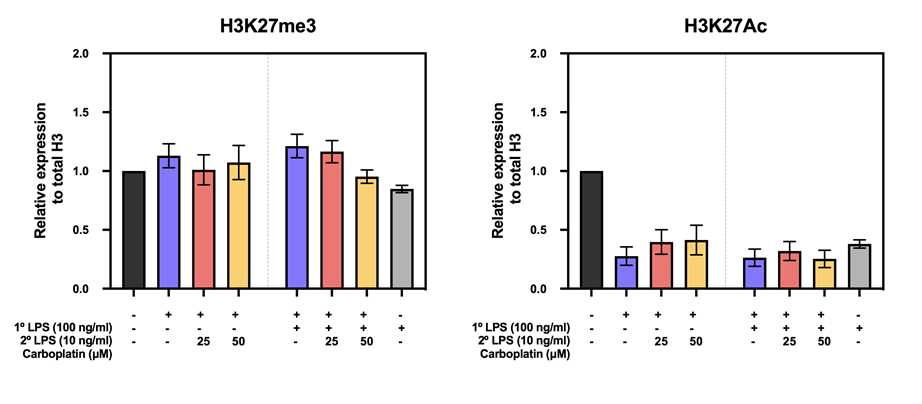


**Supplementary Fig. S7. Effects of carboplatin on global histone modification profiles of H3K27me3 and H3K27Ac.**

Histone modification profiles of LPS-primed and LPS-tolerant macrophages with or without carboplatin were examined as described in Figure 5 and the relative band intensities of H3K27me3 and H3K27Ac were measured by ImageJ analysis and normalized to the total H3 levels.

**
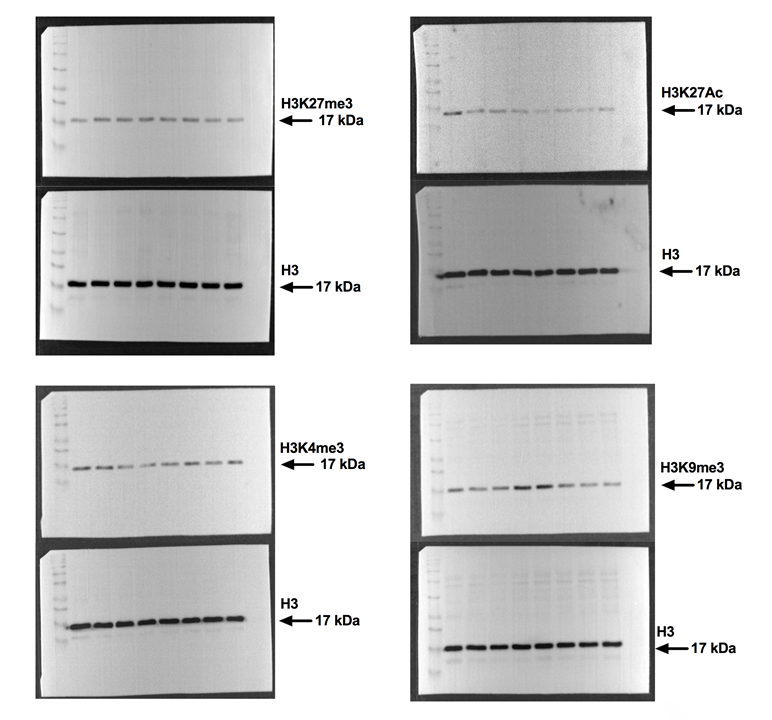
**

**Supplementary Fig. S8. Representative Western blots to investigate the effects of carboplatin on the TLR4 signaling pathway.**

Representative of unprocessed Western blots to analyze the levels of histone profiles as described in Figure 5 are shown.


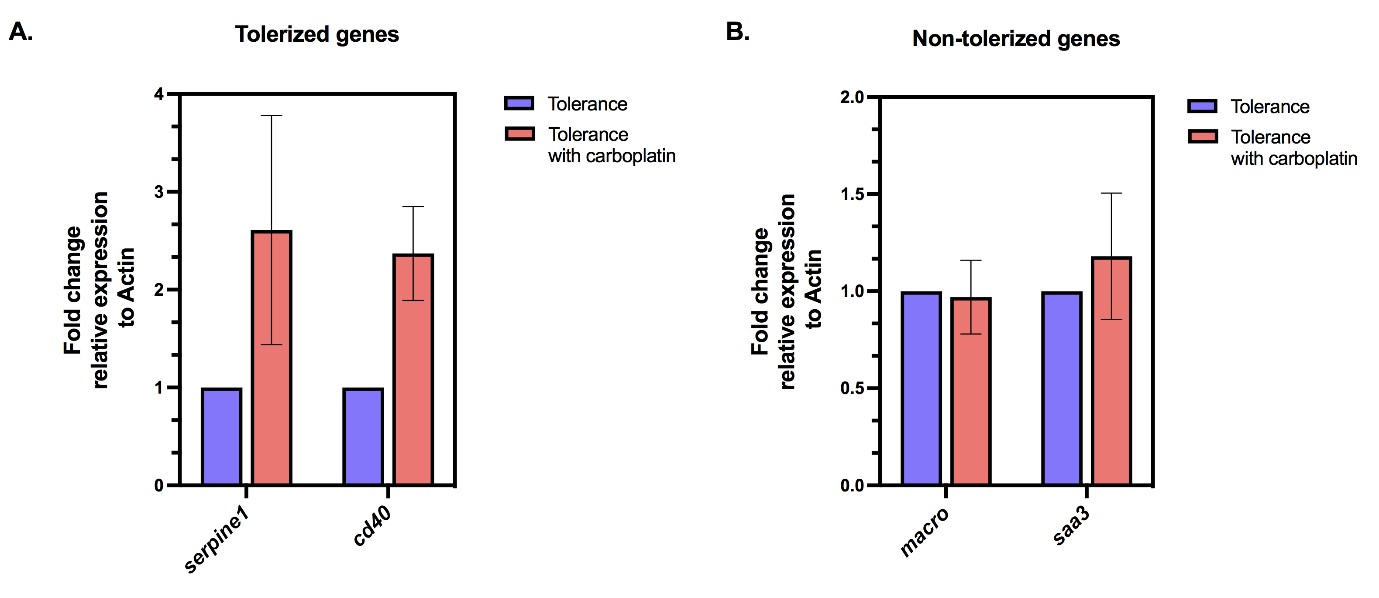


**Supplementary Fig. S9.** **Effect of carboplatin on tolerized and non-tolerized genes in LPS-tolerant macrophages.**

BMDMs were treated as indicated in Figure 1 to induce LPS-tolerant macrophages. **(A)** The relative expression level of tolerized genes; *serpine1* and *cd40*. **(B)** non-tolerized genes; *macro* and *saa3* were measured by RT-qPCR. Actin was use as control. Statistical analysis was performed by unpaired t-test, *p* > 0.05.


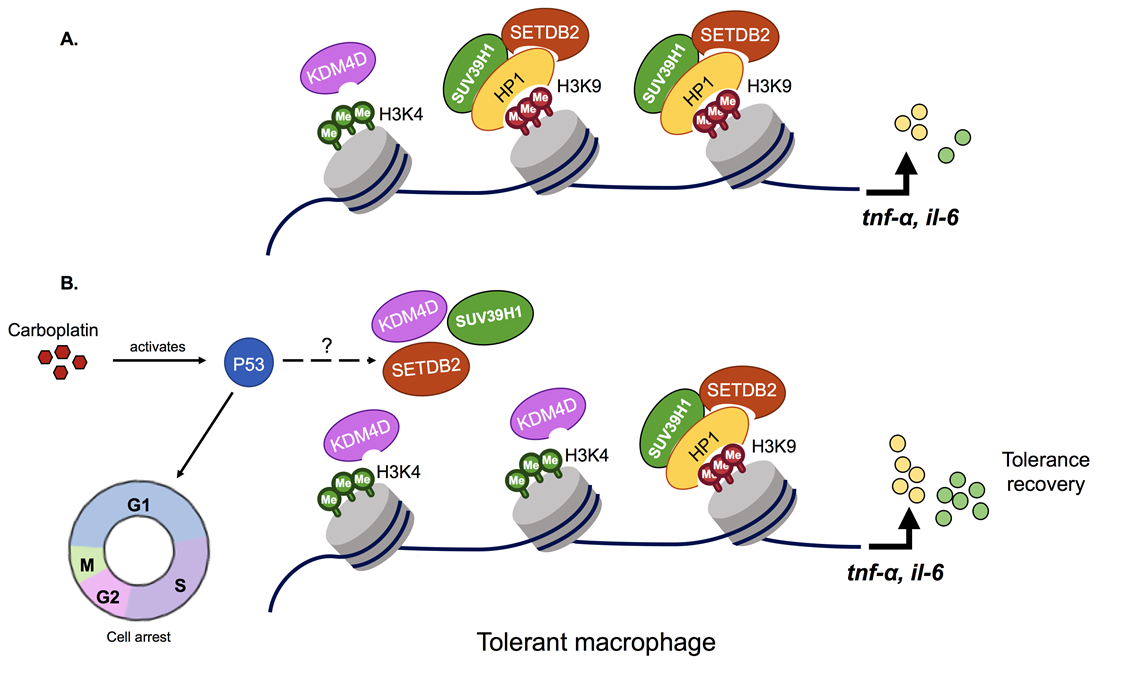


**Supplementary Fig. S10.** A proposed model how carboplatin affects LPS tolerance in macrophages. The figure is prepared by Atsadang Boonmee for this work.
